# Efficient Transformation of *Catalpa bungei* Shows *Crystal* Genes Conferring Resistance to the Shoot Borer *Omphisa plagialis*

**DOI:** 10.3389/fpls.2021.777411

**Published:** 2021-12-24

**Authors:** Fenni Lv, Peng Wang, Enliang Zhang, Lingling Ma, Lulu Gao, Rutong Yang, Qing Wang, Ya Li

**Affiliations:** Jiangsu Key Laboratory for the Research and Utilization of Plant Resources, Institute of Botany, Jiangsu Province & Chinese Academy of Sciences, Nanjing, China

**Keywords:** embryogenic callus, basal medium, plant hormone, genetic transformation, insect resistance

## Abstract

Although *Catalpa bungei* is a forest plant with considerable economic and ornamental value in China, its wood and decorative qualities are constrained by insect pests such as the shoot borer *Omphisa plagialis* (Lepidoptera). Overexpressing insect resistance genes such as *crystal* genes to develop an insect-resistant variety of *C. bungei* is an environmental and ecological approach. However, genotype limitations and low regeneration rates of embryogenic calli (EC) inhibit the development of transformation and the insect-resistant gene expression system in *C. bungei*. Here, we first established embryogenic callus induction and regeneration systems of five genotypes using mature seed and stem segment explants; the highest induction and regeneration rates of EC were 39.89 and 100%, respectively. Next, an efficient and stable *Agrobacterium*-mediated genetic transformation system was developed from EC and its positive frequency was up to 92.31%. Finally, using the transformation system, 15 and 22 transgenic *C. bungei* lines that expressed *Cry2A* and *Cry9Aa-like* were generated, respectively. These transgenic lines that exhibited significantly higher resistance to *O. plagialis* in the laboratory and field have great promise for meeting the challenge of future pest management under changing climatic conditions. Additionally, this efficient, fast, and stable transformation system could be a potential tool for gene function analysis and forest tree genetic improvement.

## Introduction

*Catalpa bungei* is both an economically important tree with high-quality wood and a magnificent ornamental tree because it is tall with a straight tree trunk and attractive and elegant flowers. It belongs to genus *Catalpa* Scop. in family *Bignoniaceae*. *C. bungei* is native to China and mainly grows in the Huanghe River and Yangtze River regions (Wang et al., 2016). *Omphisa plagialis*, the main shoot borer pest of *C. bungei*, belongs to family Pyralidae (order Lepidoptera) (Wang et al., 2012). *O. plagialis* can invade *C. bungei* young shoots from the base of the petiole into the pith of the shoots to form galls, which causes them to wither or die (Hong et al., 2016; Supplementary Figure 1). This pest is responsible for losses of up to 80% if no control measures are employed. Although spraying pesticides could control this pest, long-term pesticide use both pollutes the environment and affects human health. Genetic engineering provides potential for developing insect-resistant varieties by modifying plant genomes, specifically by expressing insect-resistant genes in plants (Bett et al., 2017). As increasing numbers of functional genes have been identified through transcriptomic and proteomic analysis (Shi et al., 2017; Xiao et al., 2020), genomic sequencing, and gene family observation (Lu et al., 2019a), establishment of an efficient genetic transformation system is desperately needed in *C. bungei*.

Developing an efficient regeneration system is important for successful genetic transformation and consequent generation of transgenic plants. Embryogenic calli (EC) have been widely used in genetic transformation of many woody plant species, such as transgenic tea (*Camellia sinensis*) (Mondal et al., 2001), European chestnut (*Castanea sativa*) (Corredoira et al., 2016), and cork oak (*Quercus suber*) (Cano et al., 2021). For *C. bungei*, several studies successfully induced calli or the EC regeneration system. For example, calli were induced using leaves as explants but did not generate EC (Lin et al., 2010). Using immature seeds as explants, two different inducing and regenerating systems of EC were developed in *C. bungei* (Jiang et al., 2010; Liu et al., 2019). However, genotype limitations and low regeneration rates of EC inhibited transformation system development in *C. bungei*.

By employing the natural gene transfer capability of *Agrobacterium tumefaciens* and *A. rhizogenes*, several genetic transformation systems were established in woody plants (Ibrahim et al., 2014; Maheshwari and Kovalchuk, 2016). A previous study established an efficient *Agrobacterium*-mediated transformation system in the commercial hybrid poplar *Populus alba* × *P. glandulosa* (Song et al., 2019). Through CRISPR/Cas9 technology and *Agrobacterium*-mediated genetic transformation, researchers generated gene-edited hemp plants (*Cannabis sativa* L.) (Zhang et al., 2021). However, to date, there have been no reports addressing the construction of genetic transformation system based on SE in *C. bungei*.

Insecticidal *crystal* genes (*Cry*) from *Bacillus thuringiensis* (Bt) bacteria are the most widely used in transgenic plants for insect control (Pardo-Lopez et al., 2013; Zhong et al., 2019). Cry toxins are highly effective against *Lepidoptera*, *Diptera*, *Hymenoptera*, and *Coleoptera* insect pests. *Cry* genes have been ectopically expressed in numerous crops, such as *Gossypium hirsutum* (Ali et al., 2016; Zubair et al., 2019), *Oryza sativa* (Pinto et al., 2013; Xu et al., 2018), *Zea mays* (Hoss et al., 2013; Hou et al., 2019), *Dioscorea esculenta* (Zhong et al., 2019), and *Solanum lycopersicum* (Koul et al., 2014), to control major insect pests, this resulted in major economic benefits.

Cry proteins have been classified into 70 subgroups. Cry2Aa, which belongs to the three-domain family, is highly effective against dipteran and lepidopteran insects (Pardo-Lopez et al., 2013). *Cry2Ab* was simultaneously expressed in cotton *via Agrobacterium*-mediated transformation to control bollworms (Siddiqui et al., 2019). Introduction of *Cry2A* in sugarcane obviously increased stem borer resistance and comparable sugar yield (Gao et al., 2018). The transgenic *Cry2A* rice effectively suppressed the occurrence of *Cnaphalocrocis medinalis*, but had no significant effects on the natural arthropod predators of *C. medinalis* (Xu et al., 2011). Cry9A is another member of the three-domain family and shows insecticidal activity against lepidopterans such as *Exorista larvarum* (Marchetti et al., 2009). Binding assays revealed that Cry9A toxins do not share receptors with Cry2A. Moreover, Cry9A toxins showed a wide spectrum of action against lepidopteran pests; therefore, Cry9A proteins have been proposed to be promising for resistance management (Li et al., 2020).

In this study, we established a novel, efficient and sustainable *A. tumefaciens*-mediated genetic transformation system of EC. We used this system to generate transgenic *C. bungei* plants expressing *Cry2A* and *Cry9Aa-like*. These transgenic plants significantly exhibited higher levels of resistance to *O. plagialis*, and therefore have great promise for meeting the challenge of future pest management under changing climatic conditions. Additionally, this efficient, fast, and generalized transformation system could be a potential tool for gene function analysis and improving wood quantity, quality, stress resistance, and rootability.

## Materials and Methods

### Plant Material

The naturally mature seeds of 13 *C. bungei* half-sib families, NJQ301–NJQ313, grown in Xuzhou, Jiangsu Province, China, were removed from the seed pods, dried, and then placed in kraft paper bags for low temperature storage. After wrapping in gauze, the stored seeds were immersed in 3 g/L carbendazim solution for 30–60 min and then rinsed under running water for 30–60 min. The seeds were then sterilized in 75% alcohol for 60 s followed by treatment with 0.1% HgCl_2_ for 18 min. The sterilized seeds were used to obtain explants for transformation studies.

All plant materials, including seedlings and regenerated shoots and plantlets, were grown in the culture room at a temperature of 25–28°C, light intensity of 1500 lx, and under a 12-h light/12-h dark photoperiod.

### Callus and Embryogenic Callus Induction Using Mature Seed Explants

The sterilized seeds of NJQ301 and NJQ302 were spread on cotyledon callusing mediums (CCMs, 30 seeds/CCM, three repetitions) and incubated in the dark to induce the primary calli (Supplementary Table 1). After 30 days of incubation on CCM, the primary calli induced from the cotyledon of mature seeds were counted.

The induced cotyledon calli were separated from the growing seedlings and transferred to embryogenic callusing mediums (ECMs, 30 calli/ECM, three repetitions) to produce EC (Supplementary Table 2). These calli were subcultured every 20 days.

### Callus and Embryogenic Callus Induction Using Stem Segment Explants

The stem segments (2–3 cm in size) were used as starting explants. The sterilized NJQ303–NJQ313 seeds were inoculated in the seedling medium [full-strength Murashige and Skoog (MS) basal medium supplemented with 30 g/L sucrose and gelled with 3 g/L gelrite, pH 5.8–6.0] for seedling culture. Stem segments were obtained from the individual NJQ303–NJQ313 seedlings and vertically inserted into the stem segment callusing mediums (SCMs, 45 stem segments/SCM, three repetitions) until the stem segment calli were induced (Supplementary Table 3). After 30 days of incubation on SCMs, the primary calli induced from the stem segments were counted.

The stem segment calli were further transferred to fresh SCMs for EC induction and subcultured every 20 days. The produced EC were separated from non-EC and multiplied further by subsequent subculturing in ECM4.

### *In vitro* Plant Regeneration and Shoot Proliferation

The actively growing EC were subsequently transferred to differentiating mediums (DMs, 30 callus clumps/DM, three repetitions) (Supplementary Table 5). The embryogenic callus clumps were subcultured every 20 days. The frequency of plant generation was calculated by dividing the number of callus clumps with differentiating shoots by the total number of callus clumps cultured. The efficiency of plant regeneration was also evaluated by scoring the mean number of shoots induced from each callus clump. The shoots obtained after 2–3 rounds of subculture were further separated and multiplied on DM11.

### Shoot Rooting and Transplantation

The multiplied shoots were then transferred to rooting mediums (RMs, 30 shoots/RM, three repetitions) (Supplementary Table 6). Then, the well-rooted plantlets were acclimatized for 5–7 days by unscrewing the cap in the culture room, and subsequently washed in water and transferred to pots containing peat: perlite (2:1). The pots were covered with transparent polybags in the greenhouse. Finally, the plants were hardened and maintained in the greenhouse or transferred to field conditions. The survival frequency of transplanted shoots was calculated by dividing the number of surviving shoots by the total number of transplanted shoots.

### *Agrobacterium* Strains, Binary Vector, and Culture

The binary vector pCAMBIA2300 was transformed into *Agrobacterium* strains EHA105 and GV3101 for optimization experiments. pCAMBIA2300 plasmid carries *uidA* (GUS) as a reporter gene and *nptII* (neomycin phosphotransferase), which is a selectable marker gene for plants and bacteria. *Cry2A* (NCBI accession NO.GQ866915) and *Cry9Aa-like* (NCBI accession NO.GQ249293) genes were inserted into pCAMBIA2300 to construct pCAMBIA2300-*Cry2A* and pCAMBIA2300-*Cry9Aa-like* plant expression vectors. EHA105 harboring pCAMBIA2300-*Cry2A* and pCAMBIA2300-*Cry9Aa-like* was used for transformation.

*Agrobacterium* culturing was conducted as described in a previous study (Maheshwari and Kovalchuk, 2016). The overnight *Agrobacterium* culture was collected by centrifugation. *Agrobacterium* suspensions of varying concentrations (OD_600_ 0.2, 0.4, 0.5, 0.6, 0.8, and 1.0) were prepared in resuspension solution (MS liquid basal medium) and used to inoculate the explants.

### Embryogenic Callus Transformation and Putative Transformant Regeneration

The fresh EC were infected by *Agrobacterium* resuspensions (OD_600_ 0.2, 0.4, 0.5, 0.6, 0.8, or 1.0; Supplementary Table 7) for varying periods of time (10, 20, or 30 min; Supplementary Table 9) with continuous shaking at room temperature. The infected calli were then washed with sterile distilled water, blotted dry on a sterile filter paper, and co-cultivated on MS basal medium at 25 ± 2°C in the dark for 24, 48, or 72 h (Supplementary Table 8).

Subsequently, the putative transformants piled up as callus clumps and selected on screening medium (DM11 added with 150 mg/L kanamycin) for 20 days, followed by DM11 containing 100 mg/L and 50 mg/L kanamycin. The kanamycin-resistant calli were transferred to DM11 with no selection pressure to induce shoot buds.

In the optimization experiments, 30 transformed embryogenic callus clumps were applied and repeated three times. The transformation frequency was calculated by dividing the number of callus clumps obtaining kanamycin resistance by the total number of infected callus clumps.

### GUS Staining Assay of Transgenic Plantlets

The GUS staining assay for transformed calli and regenerated shoots was performed according to the GUS staining kit instructions (Solarbio, G3060, China). GUS staining buffer (2.5 mL GUS buffer A, 10 μL GUS buffer B, 10 μL GUS buffer C, 2.0 mL methanol, 20 μL X-GlcA solution, 5.5 mL deionized water) added to the samples that were vacuum infiltrated for 2 min. After incubation at 37°C for 24 h, the samples were bleached by immersion in 75% ethanol, and then observed and photographed under stereo microscope (Olympus, MVX10, Japan).

### DNA Extraction and Polymerase Chain Reaction-Based Confirmation of Transgenes

DNA was extracted from leaves of regenerated plantlets using plant genomic DNA kit (DP305, Tiangen, China). The total genomic DNA quality was detected by electrophoresis on a 1.0% agarose gel. Putative transgenic plantlets that were selected by the GUS staining assay were further confirmed by polymerase chain reaction (PCR) analysis with *GUS*-specific primers (forward 5′-TGAATCCGCACCTCTGG-3′ and reverse 5′-TTCATTGTTTGCCTCCCT-3′).

### Genome Resequencing Using Next Generation Sequencing and Data Analysis

Genomic DNA was extracted from three transgenic lines and wild-type plants using a plant DNA extraction kit (Tiangen biotech, Beijing, China) according to the manufacturer’s instructions. Library construction, genome resequencing *via* next generation sequencing (NGS), and bioinformatics analysis were performed by Nextomics Bioscience Co., Ltd. (Wuhan, China). The libraries were constructed and sequenced using the MGISEQ-2000 sequencing platform (MGI, Shenzhen, China). The concentration and quality of the library were measured using an Agilent 2100 bioanalyzer (Agilent Technologies, Santa Clara, CA, United States) and Qubit 2.0 (Life Technologies, Carlsbad, CA, United States). The clean reads were compared against the *C. bungei* reference genome (Lu et al., 2019b) and the T-DNA sequence to identify junction reeds that aligned with both the reference genome sequence and the T-DNA sequence. The genome DNA-seq dataset is available at the NCBI Sequence Read Archive (SRA) with the accession number: PRJNA780358. The structures of genes located near the insertion sites (10 kb) were predicted by FGENESH.^1^

### Quantitative RT-PCR Analysis

Quantitative RT-PCR (qRT-PCR) was used to test gene expression in transgenic lines. Total RNA was extracted from the wild-type and transgenic plants using the FastPure Plant Total RNA Isolation Kit (Vazyme, RC401, China). The cDNA library was generated using the HiScript III 1st Strand cDNA Synthesis Kit (+ gDNA wiper) (R312, Vazyme, China). qRT-PCR was performed using SYBR Premix Ex Taq II (RR820, Takara, China) in the Applied Biosystems Step One Plus TM Real-Time PCR System (Applied Biosystems, United States) according to the manufacturer’s protocol.

Amplification of *Actin* (forward, 5′-GATGATGCTCCAAGA GCTGT-3′, and reverse, 5′-TCCATATCATCCCAGTTGCT-3′) was used to normalize the amount of gene-specific qRT-PCR product. The relative expression levels of *Cry2A* (forward, 5′-ACTGCACAAGACTGGCCATT-3′, and reverse, 5′-GTAGT ACCGGGAAAAGGCCC-3′) and *Cry9Aa-like* (forward, 5′-ATAGCGATGCCGTACTGCAA-3′, and reverse, 5′-TCCTG TTGTACCGATGCACC-3′) were calculated based on the C_*T*_ value from thrice-repeated real-time PCRs for each sample using the comparative ΔC_*T*_ method (Livak and Schmittgen, 2001).

### Quantification of Cry2A and Cry9Aa-Like Toxins by Enzyme-Linked Immunosorbent Assay

Leaves and stems of *Cry2A* and *Cry9Aa-like* transgenic lines were picked to measure the concentration of toxins by enzyme-linked immunosorbent assay (ELISA). A commercial ELISA kit for Cry2A (AA0741, Youlong Biotech, China) was used to measure the soluble target protein concentration in plant tissues. However, as there is no commercial ELISA Kit for Cry9A, the kit for Cry1Ab/Cry1Ac (AP003 CRBS, Envirologix QualiPlate™, United States) was used instead. All procedures were carried out in accordance with the corresponding guidelines in the users’ manuals.

### Insect Bioassays

The insecticidal efficacy of Cry2A and Cry9Aa-like toxins in transgenic *C. bungei* were analyzed on detached leaves using a larval feeding bioassay with third instar larvae of *O. plagialis*. The leaves detached from wild-type and Bt transgenic plants were used to carry out insect resistance assays, with over 30 third instar larvae of *O. plagialis* per treatment.

A ball of moist absorbent cotton was placed in a Petri dish to prevent leaf desiccation and changed daily. Insect feeding was conducted by changing fresh leaves every 2 days. The leaf consumption and larval mortality were monitored and recorded daily throughout the bioassay period (7 days), and data were obtained from three independent experiments. The corrected mortality was calculated as: corrected mortality (%) = (mortality of larvea fed transgenic leaves–mortality of larvea fed wild-type leaves)/(1–mortality of larvea fed wild-type leaves) × 100%.

## Results

### Callus Initiation and Embryogenic Callus Induction of *Catalpa bungei*

**FIGURE 1:**
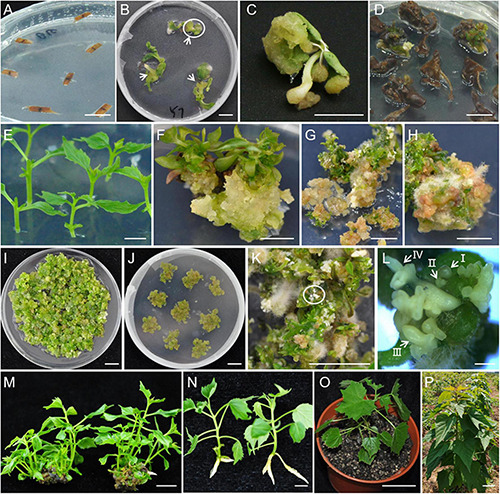
Genetic transformation system of *Catalpa bungei*. **(A–C,E,F)** The explants of mature seeds **(A)** and stem segments **(E)** were employed to induce calli [**(B,F)**, respectively]. The white arrows indicate the induced callus in panels **(B,C)** was the enlarged close-up photos on the circled portion in panel **(B)**. **(D,G,H)** The two types of calli were transferred to embryogenic callusing medium to produce embryogenic calli (EC), respectively. The EC were further proliferated **(I)** and piled up as callus clumps **(J)** to regenerate somatic embryos **(K,L)**, and shoots **(M)**. **(L)** Shows enlarged close-up photos of the circled portion in panel **(K)**. I, II, III, and IV in panel **(L)** indicate the globular somatic embryo, heart-shaped somatic embryo, torpedo-shaped somatic embryo, and cotyledonary somatic embryo, respectively. After rooting of regenerated shoots **(N)**, plantlets were acclimatized and hardened, and then grown in the greenhouse **(O)** and field **(P)**. Bars = 1 cm **(A–K,M,N)**, 1 mm **(L)**, 5 cm **(P)**, and 10 cm **(P)**.

Almost 94–98% of mature seeds from the half-sib family NJQ301 on CCM16 induced yellowish-white calli, which was the highest frequency among the 17 CCMs (Figures 1A–C and Supplementary Table 1). In addition, we found that sucrose concentration affected callus induction (Supplementary Figure 2). The amount of callus was the largest when sucrose was 30 g/L, followed by 60 g/L, and then 90 g/L. For callus quality, as the sucrose concentration increased, the degree of callus browning gradually deepened (Supplementary Figure 2).

These calli were transferred to ECMs and the statistics showed that ECM16 was the most suitable medium for EC induction because approximately 10.61% of brown calli induced EC (Figure 1D and Supplementary Table 2). Additionally, the EC transferred to ECMs with MS basal medium were highly proliferative, especially ECM4 (2–3 times the initial amount after 20 days) (Figure 1I). Mature seeds from NJQ302 were firstly inoculated on CCM16 followed by ECM16, and the EC induction rate was 3.51% after 150–180 days (Supplementary Figure 3).

Because of the low induction frequency and how time-consuming it was for EC to be initiated from mature seeds, we developed another induction protocol for EC with stem segments. Three individuals from NJQ305, NJQ308, and NJQ313 produced EC on SCMs after 60–90 days (Supplementary Table 3 and Supplementary Figure 3). These three individuals were subsequently propagated and the statistics showed that 39.89, 29.51, and 36.99% of stem segments generated EC on SCM3, SCM6, and SCM11, respectively (Figures 1E–H and Supplementary Table 4).

### *In vitro* Regeneration System of *Catalpa bungei*

The subsequent transfer of NJQ301 EC to DMs, piling up as callus clumps (Figure 1J), led to the emergence of somatic embryos (SE) and shoot buds (Figure 1K). The SE directly developed into shoots through four periods: globular, heart-shaped, torpedo-shaped, and cotyledonary embryoid periods (Figure 1L). In addition, among the 11 DMs, the addition of cytokinin and auxin to MS basal medium did not have any effect on shoot emergence or plant regeneration efficiency (Supplementary Table 5). Interestingly, changing the basic medium to Driver and Kuniyuki Walnut medium (DKW) yielded 100% regeneration efficiency. An average of 5–10 shoots developed from each EC clump, and these shoots were allowed to grow further and multiplied on DM11 (Figure 1M). The maximum rooting efficiency of the regenerated shoots was 90.88% in RMs (Figure 1N and Supplementary Table 6). Indole-3-butytric acid (IBA) had a better response compared with 1-Naphthylacetic acid (NAA) as a cutting rooting regulator in the medium combinations. The well-rooted plantlets showed a 90–95% survival frequency when transferred to greenhouse and field conditions (Figures 1O,P).

**FIGURE 2:**
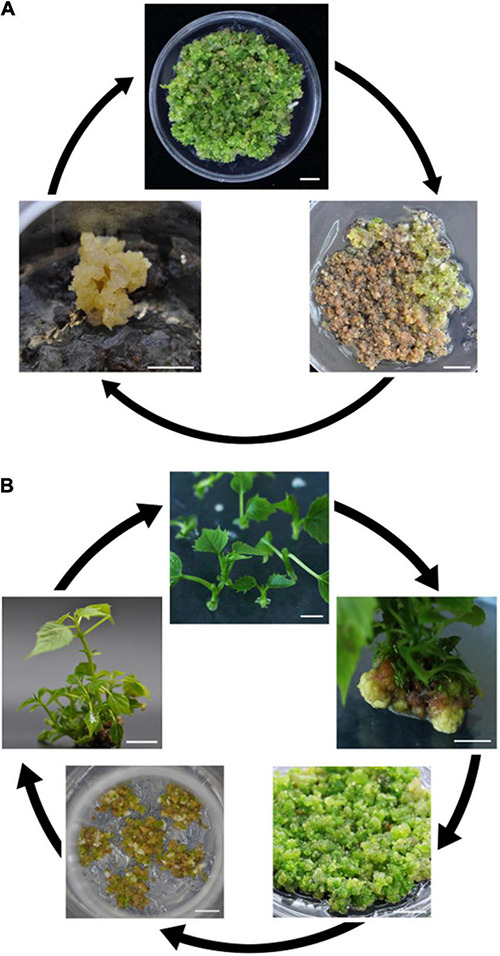
Illustration representing the recycling of two types of embryogenic calli (EC) from mature seeds **(A)** and stem segments **(B)**. Bars = 1 cm **(A,B)**.

During the proliferation of EC, it was observed that multiple long-term subcultures resulted in decreased or even lost regeneration of EC, and the calli turned brown. Miraculously, from the brown EC, a new EC arose, although this process took a long time (Figure 2A). For stem EC, to avoid activity decrease, the regenerated plantlets were employed to induce new EC (Figure 2B).

### *Agrobacterium*-Mediated Transformation of Embryogenic Calli

**FIGURE 3:**
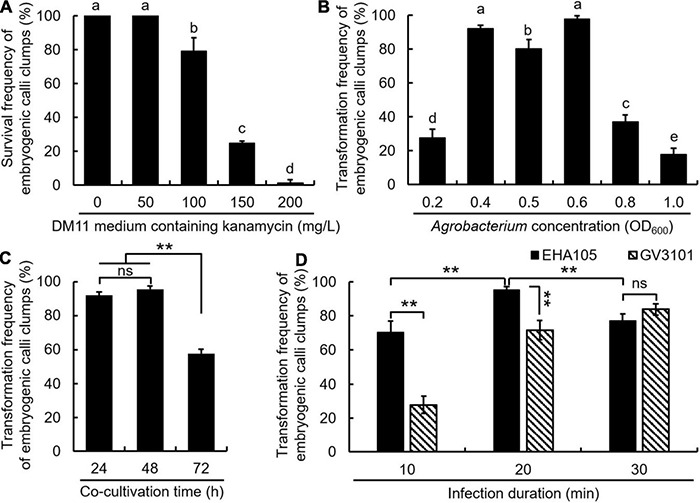
Evaluation of five factors affecting the transformation efficiency. The survival frequency statistical results **(A)** of the embryogenic calli (EC) transferred to DM11 medium containing different concentrations of kanamycin. Bacterial concentration **(B)**, co-cultivation time **(C)**, and *Agrobacterium* strain and infection duration **(D)** were found to differently affect the transformation frequency of embryogenic callus clumps. Results are presented as means and standard errors from three independent experiments, each with 30 embryonic callus clumps. Different lowercase letters (a–e) among the levels of each factor indicate the statistical differences between them (*P* < 0.05, one-way ANOVA). **p < 0.01, ns indicates no significant difference (Student’s *t*-test).

The NJQ301 EC were used as explants for *Agrobacterium*-mediated transformation study. In this process, five factors that affect transformation efficiency were tested, including kanamycin concentration, bacterial concentration, co-cultivation time, *Agrobacterium* strain, and infection duration. As shown in Figure 3A and Supplementary Figure 4, the NJQ301 EC clumps transferred to DM11 with 50 mg/L kanamycin had 100% survival frequency, but exhibited growth retardation compared with 0 mg/L. Moreover, 100, 150, and 200 mg/L kanamycin, respectively, exhibited 79, 25, and 0% survival frequency. Therefore, DM11 with 150 mg/L kanamycin was selecting furthered transgenic EC.

With the increase of bacterial concentration, the transformation frequency continued to increase, reaching the highest value of 97% at OD_600_ = 0.6, and then decreased at OD_600_ ≥ 0.8 (Figure 3B and Supplementary Table 7). Therefore, 6 × 10^8^ cells/mL was optimal bacterial concentration for the EC-mediated transformation in *C. bungei*. The extension of co-cultivation period to 72 h significantly reduced the transformation frequency; thus, 48 h was most effective (Figure 3C and Supplementary Table 8). The *Agrobacterium* strain EHA105 was found to be comparatively more efficient than GV3101 for transforming *C. bungei* EC, and an infection time of 20 min was found to be optimal when immersed in EHA105 resuspensions (Figure 3D and Supplementary Table 9).

### Transgenic Plantlet Regeneration and Screening

To efficiently screen out the transformed EC while reducing the suppression of kanamycin on EC growth, we developed a process of three levels of kanamycin selection of transgenic EC. Therefore, after the first round of selection on DM11 containing 150 mg/L kanamycin, the transformed EC were then subsequently selected on DM11 containing 100 and 50 mg/L kanamycin in turn. These kanamycin-resistant EC were then cultured on kanamycin-free DM11 to stimulate regeneration.

**FIGURE 4:**
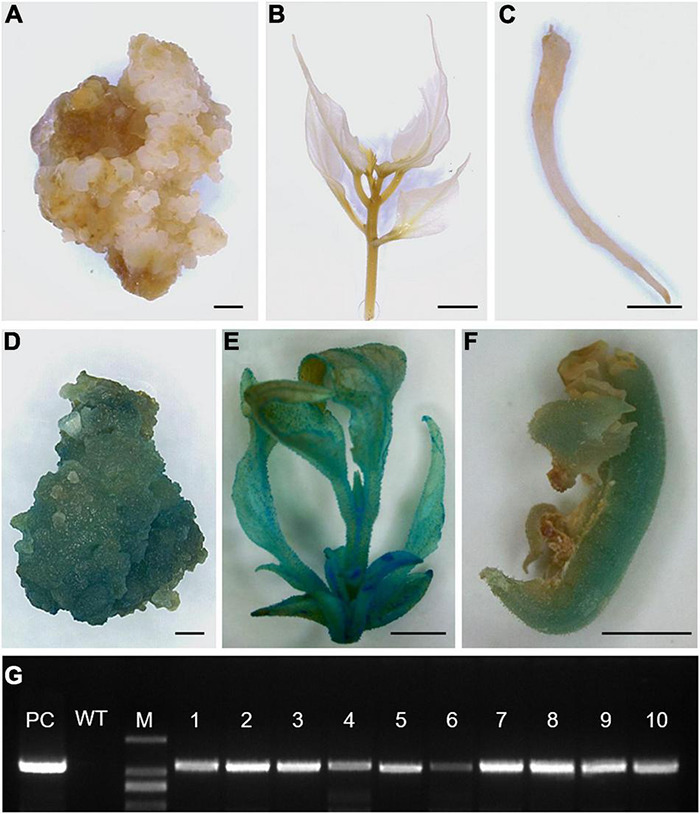
Detection of the reporter gene *GUS* in transgenic embryogenic calli (EC) and plantlets. **(A–F)** GUS staining analysis of wild-type (no blue staining) and transgenic (blue) EC **(A,D)**, cutting shoots **(B,E)**, and roots **(C,F)**. **(G)** PCR detection of the reporter gene *GUS* in transgenic plantlets, with pCAMBIA2300 vector as a positive control (PC) and wild-type plants as a negative control (WT). Bars = 0.5 mm **(A,D)**, 0.5 cm **(B,C,E,F)**.

After subculturing 2–3 times on DM11, the kanamycin-resistant EC exhibited shoot bud emergence. The transgenic nature of shoots and transformed EC were confirmed by histochemical staining of GUS activity (Figures 4A–F). The positive plants in GUS detection were further confirmed for transgene through PCR amplification of the GUS reporter gene (Figure 4G). In this study, 284 regenerated individuals were detected and the positive frequency was up to 92.31% (Supplementary Table 10).

**FIGURE 5:**
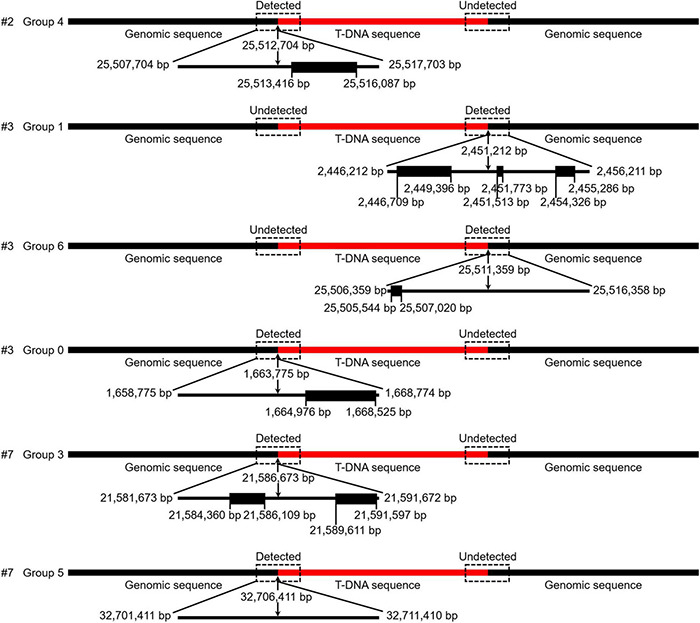
The detection results of T-DNA insertion sites obtained *via* next generation sequencing (NGS). Detected/Undetected indicates that the junction reads (reads containing both T-DNA and flanking genomic sequences) were identified or not identified in NGS data. The genes (black boxes) located near the insertion sites (10 kb) were predicted by the online program FGENESH.

To further verify the T-DNA sequence was inserted into *C. bungei* genome, we performed the genome sequencing of three positive lines (#2, #3, and #7 in Figure 4G) and the wild-type plants. More than 130 million clean reads were obtained, respectively, from the raw reads for transgenic and wild-type plants, corresponding to 25–31 × coverage of the *C. bungei* reference genome (Lu et al., 2019b). 91.83–93.20% of the sequencing data had Phred-like quality scores ≥30, indicating that the data were high quality (Supplementary Table 11). After sequence alignment, one junction read in the #2 genome, three junction reads in the #3 genome, and two junction reads in the #7 genome were identified (Supplementary Table 12 and Figure 5). While, T-DNA sequence were not detected in the wild-type genome. These results indicated that there is only one T-DNA insertion site in the #2 genome, multiple T-DNA insertion sites in the #3 and #7 genomes, and showed that this protocol is a stable genetic transformation system.

### Evaluation of Bt *Catalpa bungei* Entomotoxic Effects on *Omphisa plagialis*

To generate *O. plagialis*-resistant *C. bungei*, we constructed pCAMBIA2300-*Cry2A* and pCAMBIA2300-*Cry9Aa-like* plant expression vectors and transformed the two Bt genes into the *C. bungei* genome using the *Agrobacterium*-mediated transformation protocol described herein. A total of 37 independent Bt transformed lines, including 15 *Cry2A* transgenic lines and 22 *Cry9Aa-like* transgenic lines were generated using the genetic transformation protocol described herein.

**FIGURE 6:**
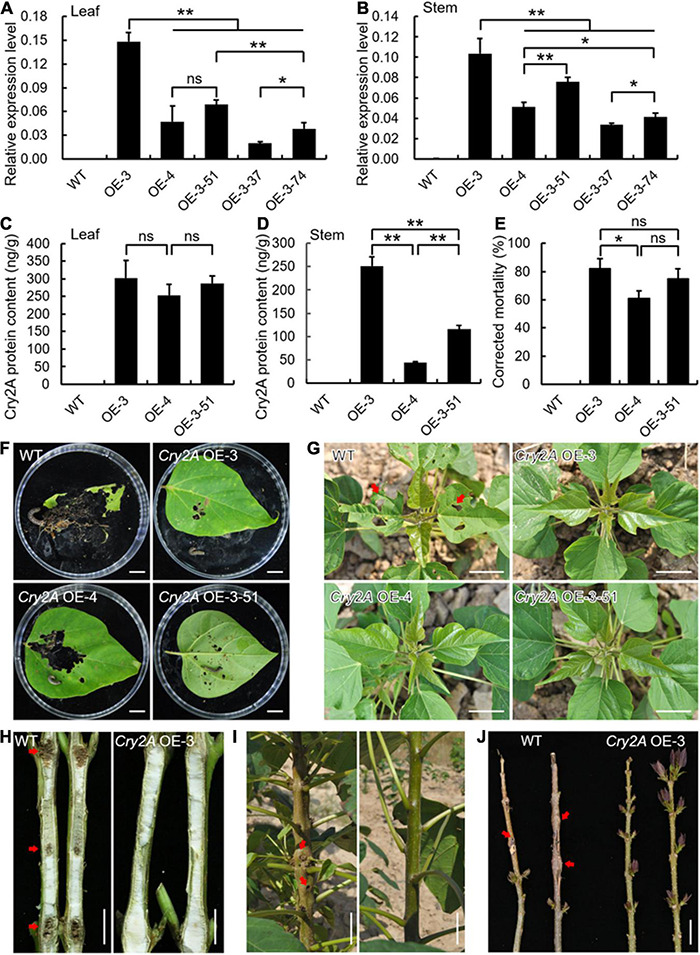
Ectopic expression of *Cry2A* enhanced insect resistance to *Omphisa plagialis* in transgenic *Catalpa bungei* lines. **(A,B)** Expression analysis of *Cry2A* in wild-type (WT) and transgenic lines (OE-3, OE-4, OE-3-51, OE-3-37, OE-3-74) in leaves **(A)** and stems **(B)**. qRT-PCR data were normalized using the internal control expression of *Actin*. Error bars represent the standard deviation of expression from three independent biological replicates. **(C,D)** Detection of Cry2A toxin protein content of WT and three selected transgenic lines in leaves **(C)** and stems **(D)**. Error bars represent the standard deviation of protein content from three independent biological replicates. **(E)** Corrected mortality statistics of WT and *Cry2A* transgenic lines. Results are presented as means and standard errors from three independent experiments, each with over 30 larvae of *Omphisa plagialis*. **(F)** Leaf phenotypes of WT and *Cry2A* transgenic lines damaged by insects in the laboratory. **(G–J)** Leaf and stem phenotypes of WT and *Cry2A* transgenic lines damaged by insects in the field. Bars = 1 cm **(F,H)**, 5 cm **(G,I,J)**. In panels **(A–E)**, *p < 0.05, **p < 0.01, ns indicates no significant difference (Student’s *t*-test).

To assess the efficiency of transgenic Bt line insect resistance, we first observed the expression pattens of *Cry2A* using qRT-PCR analysis. *Cry2A* was ectopically expressed to varying degrees in leaves and stems of five arbitrarily selected lines, whereas untransformed control plants failed to detect *Cry2A* (Figures 6A,B). The *Cry2A* transcript levels in the OE-3, OE-4, and OE-3-51 lines were relatively higher among the five lines and were selected for further examination. Detection of Cry2A content in these three transgenic lines based on protein level showed that the Cry2A toxin protein present in transgenic leaves ranged from 253.45 to 302.58 ng/g, and 44.15 to 251.47 ng/g in transgenic stems with similar patterns of *Cry2A* transcript levels (Figures 6C,D). It is worth noting that the *Cry2A* OE-3 transgenic line exhibited the most Cry2A content in both transcript and protein levels in the leaves and stems. Moreover, the insect bioassays revealed that the corrected mortality of *O. plagialis* was as high as 81.85% after being fed *Cry2A* OE-3 leaves, whereas the insects fed wild-type leaves grew well and were fat and strong (Figures 6E,F).

In wild-type plants in the field, the leaves were seriously damaged by the larvae (Figure 6G), and shoot piths were bored and formed galls in multiple sites (Figures 6H–J). In contrast, the damaged leaves in *Cry2A* transgenic lines were relatively reduced (Figure 6G), and the bored sites and galls also decreased (Figures 6H–J), exhibiting insect resistance. Statistically, 2.5% of *Cry2A* OE-3 plants compared with 45.0% wild-type plants were bored by *O. plagialis* (Supplementary Figure 5).

**FIGURE 7:**
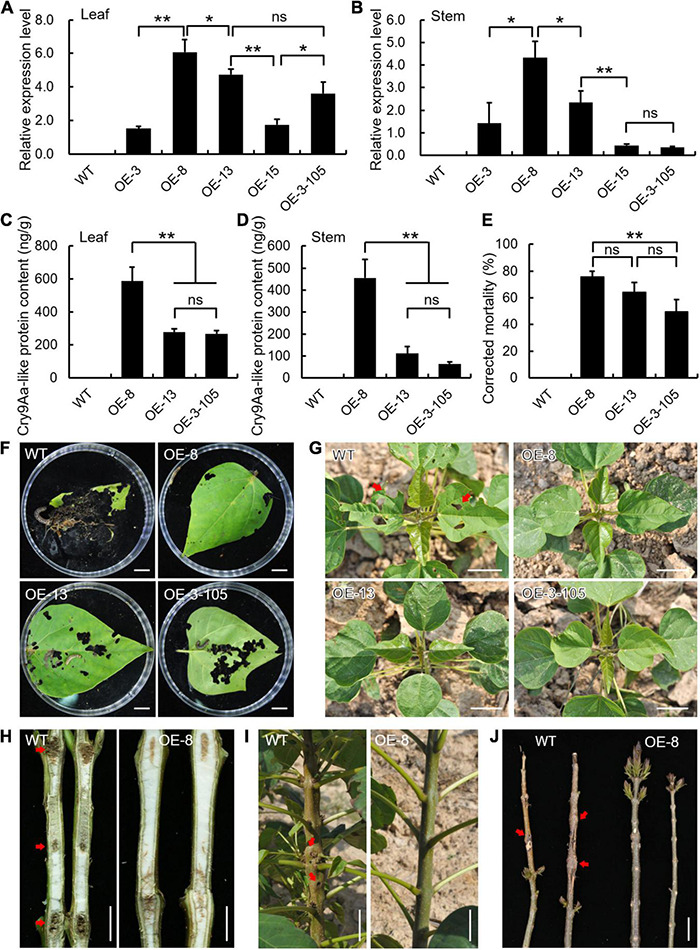
Ectopic expression of *Cry9Aa-like* in *Catalpa bungei* exhibited enhanced resistance to *Omphisa plagialis*. **(A,B)** Expression analysis of *Cry9Aa-like* in wild-type (WT) and transgenic lines (OE-3, OE-8, OE-13, OE-15, OE-3-105) in leaves **(A)** and stems **(B)**. qRT-PCR data were normalized using the internal control expression of *Actin*. Error bars represent the standard deviation of expression from three independent biological replicates. **(C,D)** Detection of Cry9Aa-like toxin protein content of WT and three selected transgenic lines in leaves **(C)** and stems **(D)**. Error bars represent the standard deviation of protein content from three independent biological replicates. **(E)** Corrected mortality statistics of WT and *Cry9Aa-like* transgenic lines. Results are presented as means and standard errors from three independent experiments, each with over 30 larvae of *O. plagialis*. **(F)** Leaf phenotypes of WT and *Cry9Aa-like* transgenic lines damaged by insects in the laboratory. **(G–J)** Leaf and stem phenotypes of WT and *Cry9Aa-like* transgenic lines damaged by insects in the field. Bars = 1 cm **(F,H)**, 5 cm **(G,I,J)**. In panels **(A–E)**, *p < 0.05, **p < 0.01, ns indicates no significant difference (Student’s *t*-test).

Coincidentally, in *Cry9Aa-like* transgenic lines, a series of consistent results was obtained in terms of transcript level, toxin protein content, and insect bioassays. As shown in Figure 6, *Cry9Aa-like* transcripts diversely accumulated in transgenic leaves and stems (Figures 7A,B). Accordingly, three transgenic lines with relatively higher expression levels (OE-8, OE-13, and OE-3-105) were chosen for further examination. Cry9Aa-like toxin protein content ranged from 267.26 to 588.95 ng/g in leaves and 62.54 to 455.67 ng/g in stems (Figures 7C,D). *Cry9Aa-like* OE-8 had the highest toxin protein content with the strongest insect resistance, and the corrected mortality was as high as 75.19% (Figures 7E,F). The damage degree of transgenic leaves and shoot piths was reduced, although the petiole base exhibited bored marks from larvae (Figures 7G–J). Additionally, the field statistics showed that 9.5% of *Cry9Aa-like* OE-8 plants were damaged, which was much lower than that of the wild-type plants (Supplementary Figure 5).

## Discussion

*Catalpa bungei* is a precious tree species, with high-quality wood and high ornamental value. However, research of its gene functions has significantly lagged behind *Populus*. A very important reason for this lack of research is the lack of an efficient regeneration and genetic transformation system of *C. bungei*. Overcoming this obstacle can promote functional genomics studies to transform the accumulated information into basic and applied research. Here, we reported a comprehensive, highly efficient, and generalized regeneration and transformation system of *C. bungei* for the first time. We created 27 *O. plagialis*-resistant germplasms by applying this system.

### Multiple Genotypes and Multiple Explant Types Were Involved in the Embryogenic Calli Protocol of *Catalpa bungei*

Embryogenic calli induction and regeneration is a process that limits the establishment of efficient genetic transformation systems (Palomo-Rios et al., 2021). Genotype is considered the determinant factor in the success of this process in plant species because protocols suitable for one embryogenesis line or clones may not necessarily apply to others (Petri and Burgos, 2005). Only one or two EC genotypes were developed in previous genetic transformation protocols of woody plants, such as *Paulownia elongata* (Bajaj et al., 2021), olive (*Olea europaea* L.) (Narvaez et al., 2019; Palomo-Rios et al., 2021), Cork oak (*Quercus suber* L.) (Alvarez et al., 2004; Cano et al., 2021), European chestnut (Corredoira et al., 2016), and tea (*Camellia sinensis*) (Mondal et al., 2001). In this study, we obtained five EC genotypes, including two types of EC initiated from mature seed explants and three types from stem segment explants, which indicated that our constructed EC protocol was independent of genotype; therefore, it might be able to be extended to elite cultivars.

The transformed explants ranged from shoot apices (Ceasar et al., 2017), stem internodes (Confalonieri et al., 2000), axillary buds (Maheshwari and Kovalchuk, 2016), and leaves (Confalonieri et al., 1994; Yang et al., 2016; Song et al., 2019) to EC and somatic embryos from specific starting explants (Corredoira et al., 2016; Sood et al., 2020; Cano et al., 2021). First, the use of vegetative tissues and organs, such as leaf and stem internodes, had the disadvantages of chimeric transgenic plant production and low transformation efficiency. Second, the starting explants applied to initiate EC involve immature inflorescences (Wang et al., 2011), immature embryos (Konagaya et al., 2020; Ma et al., 2020), and leaves (Sane et al., 2012). The success of the studies using immature zygotic embryos or inflorescences depends on the embryo or inflorescence quality, which can cause problems and disadvantages, including that it is difficult to ensure ease of their availability throughout the year, especially for forest trees. Therefore, we used mature seeds as explants for EC because large quantities can be easily obtained and kept viable for unlimited access throughout the year. Furthermore, because of the low induction frequency and how time-consuming it was for EC to be initiated from mature seeds, we developed another EC induction protocol using stem segments excised from micropropagated plantlets as starting explants. Three genotypes of EC were obtained after 90–100 days using stem segments, and the induction rate was up to 39.89%; the higher the induction rate, the less induction time and more uniform genotypes compared with mature seeds.

In particular, in the process of long-term and recurrent proliferation, EC inevitably browned, or even died, which seriously affected the transformation efficiency. Amazingly enough, EC could be renewable after browning, and re-acquired sufficient capabilities for regeneration and transformation.

### The Established *Agrobacterium*-Mediated Transformation System Provides a Powerful Tool for Gene Functional Analysis in *Catalpa bungei*

Genetic transformation efficiency is the ultimate and utmost important parameter of genetic transformation protocols. In woody plants, the transformation frequency varies, from 4% obtained by *A. tumefaciens*-mediated transformation with embryogenic masses of cork oak trees (Alvarez et al., 2004), 7% by *A. tumefaciens*-mediated transformation with internodal stem segments from white poplar (*Populus alba* L.) plantlets (Confalonieri et al., 2000), to 32.2% by *Agrobacterium*-mediated transformation with juvenile leaf explants in hybrid poplar *P. alba* × *P. glandulosa* Uyeki (Song et al., 2019). We optimized the *Agrobacterium*-mediated transformation system of EC and improved the positive frequency to 92.31% with our protocol. This system was divided into five major processes: primary callus induction, embryogenic callus induction and proliferation, transformation with *Agrobacterium*, transgenic plantlet differentiation and regeneration, and transgenic plant transplantation (Supplementary Figure 6).

The efficiency of the *Agrobacterium*-mediated transformation was reported to be affected by multiple factors, including explant type, *Agrobacterium* strain, selection regime, and regeneration system (Sood et al., 2020). The high efficiency of our protocol should be attributed to the following aspects. First, freshly developed EC were used as target material and were considered the most critical factor because they have higher transformation and regeneration potential. Second, the *Agrobacterium* strain, bacterial concentration, infection duration, co-cultivation period, selection regime, medium formula, hormone combination, and hormone concentration were optimized. Third, three levels of kanamycin selection of transgenic cells effectively eliminated the non-transformed EC and increased the positive frequency of regenerated plantlets. Additionally, a combination of PCR detection and GUS assays ensured that all transgenic plantlets were obtained.

Finally, by optimizing all of the various affected factors, this study produced a highly efficient, reliable, and reproducible *A. tumefaciens*-mediated genetic transformation system. In addition to the *Cry2A* and *Cry9Aa-like* Bt genes, we also transferred adventitious root formation-related genes and salt stress response-related genes by employing this system. The function and molecular mechanism of these genes are currently being analyzed in *C. bungei*. The amplification of *GUS* exhibited successful integration of target genes into the *C. bungei* plantlet genome. The appearance of blue staining in EC, cut shoots, and roots, and the transcript accumulation of *Cry2A* and *Cry9Aa-like* in leaves and stems confirmed the successful expression of target genes.

### *Cry2A* Transgenic *Catalpa bungei* Exhibited Better Resistance to *Omphisa plagialis*

A considerable number of crops have been reported to effectively control target insects by ectopically expressing Bt toxins (Hoss et al., 2013; Pardo-Lopez et al., 2013; Shu et al., 2013; Schunemann et al., 2014; Tian et al., 2017; Gao et al., 2018). Thus, Bt transgenic crops are considered a cornerstone in integrated pest management (Xu et al., 2020). In particular, Bt transgenic cotton has been commercialized. In forest trees, there are relatively fewer studies on insect pest resistance by expression of Bt genes. However, *Cry3Bb* was introduced into the poplar plastid genome to control *Plagiodera versicolora* (Xu et al., 2020). *Cry3A* transgenic poplar (BGA-5) significantly increased the total mortality of the target pest *P. versicolora*, but no significant effect was detected on the non-target pest *Clostera anachoreta* under laboratory conditions (Zhang et al., 2011). *Cry1Ac–Cry3A* transgenic poplar exhibited high resistance to the first instar larvae of *Hyphantria cunea* and *Micromelalopha troglodyta*, and the first and second instar larvae and adults of *P. versicolora* (Ren et al., 2021). Moreover, previous studies provided evidence that Bt transgenic poplar or crops did not pose an ecological risk and did not affect the microbial community structure or functional diversity (Wang et al., 2015; Yang et al., 2015, 2018; Zou et al., 2016; Zuo et al., 2018; Ali et al., 2020).

Damage caused by *O. plagialis* can severely reduce plantation growth, resulting in substantial stand losses to the wood industry. However, to our knowledge, there are no published reports that describe transgenic *C. bungei* with Bt toxins and Bt toxicity on *O. plagialis*. This study showed that transgenic *C. bungei* overexpressing *Cry2A* and *Cry9Aa-like* can prevent *O. plagialis* infestation. Additionally, compared with Cry9Aa-like, Cry2A was more toxic to this insect pest. Coincidentally, in a previous study, Cry1Ac, Cry1Fa, Cry1Ca, and Cry2Aa were shown to have varying toxicity to both *Anticarsia gemmatalis* and *Chrysodeixis* (*Pseudoplusia*) *includens*, and distinct binding sites in the midguts of soybean pests (Bel et al., 2017). Therefore, Cry2A-binding sites may not be shared with Cry9Aa-like in *O. plagialis*. Our research broadens the knowledge regarding the insect resistance spectrum of Bt genes and provides a reference for research and use of forest trees that overexpress Bt genes for insect resistance.

## Data Availability Statement

The datasets presented in this study can be found in online repositories. The names of the repository/repositories and accession number(s) can be found in the article/Supplementary Material.

## Author Contributions

PW, YL, and FL designed the experiments. FL, PW, and EZ executed the experiments, analyzed the data, and assembled the figures. LM and LG performed subculturing of calli. RY and QW performed regenerated shoots transplantation. FL and PW wrote the manuscript. All authors read and approved the final manuscript.

## Conflict of Interest

The authors declare that the research was conducted in the absence of any commercial or financial relationships that could be construed as a potential conflict of interest.

## Publisher’s Note

All claims expressed in this article are solely those of the authors and do not necessarily represent those of their affiliated organizations, or those of the publisher, the editors and the reviewers. Any product that may be evaluated in this article, or claim that may be made by its manufacturer, is not guaranteed or endorsed by the publisher.

## Footnotes

1
http://www.softberry.com/berry.phtml?topic=fgenesh&group=programs&subgroup=gfind
