# Supplementary material for: Efficient Transformation of Catalpa bungei Shows Crystal Genes Conferring Resistance to the Shoot Borer Omphisa plagialis
Source: Front Plant Sci. 2021 Dec 24;12:777411. doi: 10.3389/fpls.2021.777411 (PMC8739885; doi:10.3389/fpls.2021.777411)
Supplement: Supplementary file 1 [file Data_Sheet_1.pdf]

Supplementary Table 1. Composition of medium tested for callus induction from mature *Catalpa bungei* seeds.

**Cotyledon callusing medium**	**Components**								**Frequency (%)**	**Significance***
	**Basic medium**	**TDZ (mg/L)**	**ZT (mg/L)**	**IAA (mg/L)**	**2,4-D (mg/L)**	**6-BA (mg/L)**	**NAA (mg/L)**	**Sucrose (g/L)**		
CCM1	MS							30	0	g
CCM2	1/2MS	0.30		0.30				30	45.04 ±8.52	f
CCM3	1/2MS	0.30		0.50				30	53.57 ±7.25	d
CCM4	1/2MS	0.30		0.70				30	50.83 ±3.82	e
CCM5	1/2MS	0.30		0.90				30	44.05 ±7.79	f
CCM6	1/2MS	0.30		0.50	0.05			30	58.33 ±6.29	c
CCM7	1/2MS	0.30		0.70	0.05			30	70.99 ±7.03	b
CCM8	1/2MS	0.30		0.90	0.05			30	63.45 ±6.40	b
CCM9	1/2MS		0.30	0.30				30	53.41 ±7.41	d
CCM10	1/2MS		0.30	0.50				30	72.11 ±4.42	b
CCM11	1/2MS		0.30	0.70				30	90.84 ±2.20	a
CCM12	1/2MS		0.30	0.90				30	65.03 ±9.69	b
CCM13	1/2MS		0.20	0.50				30	88.40 ±4.06	a
CCM14	1/2MS		0.20	0.50				60	57.00 ±7.55	c
CCM15	1/2MS		0.20	0.50				90	87.73 ±4.86	a
**CCM1**6	**MS**					**0.50**	**0.20**	**30**	**96.41 ±1.84**	**a**
CCM17	MS					0.70	0.30	30	94.70 ±2.62	a

MS, full-strength Murashige and Skoog basal medium; 1/2MS, half-strength MS; TDZ, Thidiazuron; ZT, Zeatin; IAA, Indole-3-acetic acid; 2,4-D, 2,4-Dichlorophenoxyacetic acid; 6-BA, 6-Benzylaminopurine; NAA, 1-Naphthylacetic acid.
The CCMs were gelled with 3 g/L gelrite, pH 5.8-6.0.
30 mature seeds of NJQ301were inoculated for each type of CCMs, repeated three times.
* Different lowercase letters (a, b, c, d, e, f, and g) indicate the statistical differences between CCMs ($P < 0.05$).

Supplementary Table 2. Composition of medium tested for embryogenic callus induction of *Catalpa bungei*.

Embryogenic callusing medium	Components					Frequency (%)	Significance*
	Basic medium	6-BA (mg/L)	NAA (mg/L)	IBA (mg/L)	IAA (mg/L)		
ECM1	MS					0	d
ECM2	MS	0.30	0.10			2.22 ±0.92	b
ECM3	MS	0.60	0.10			3.50 ±0.19	b
ECM4	MS	0.60	0.15			3.58 ±0.22	b
ECM5	MS	0.75	0.15			3.54 ±0.19	b
ECM6	MS	0.60	0.20			3.58 ±0.21	b
ECM7	MS	1.00	0.20			1.11 ±0.92	c
ECM8	MS	2.00	0.30			0	d
ECM9	MS	3.00	1.00			0	d
ECM10	MS	4.00	1.50			0	d
ECM11	MS	0.50		0.05		0	d
ECM12	MS	1.00		0.05	0.10	1.11 ±0.93	c
ECM13	MS	0.10		0.50		0	d
ECM14	MS	0.50		1.00		1.15 ±0.99	c
ECM15	MS	1.00		1.50		0	d
**ECM16**	**DKW**	**0.60**	**0.15**			**10.61 ±0.56**	**a**

MS, Murashige and Skoog basal medium; DKW, Driver and Kuniyuki walnut basal medium; 6-BA, 6-Benzylaminopurine; NAA, 1-Naphthylacetic acid; IBA, Indole-3-butytric acid; IAA, Indole-3-acetic acid.

The ECMs were added with 30g/L sucrose and gelled with 3 g/L gelrite, pH 5.8-6.0.
30 calli developed from mature seeds were inoculated for each type of ECMs, repeated three times.
* Different lowercase letters (a, b, c, and d) indicate the statistical differences between ECMs ($P < 0.05$).

Supplementary Table 3. Composition of medium tested for callus induction from stem segments of *Catalpa bungei*.

**Stem segment callusing medium**	**Components**				***C. bungei* half-sib families**	**No. of stem segments inoculated**	**No. of embryogenic calli developed**
	**Basic medium**	**6-BA (mg/L)**	**IBA (mg/L)**	**NAA (mg/L)**			
SCM1	MS			0.1	NJQ303	45	0
SCM2	MS		0.1		NJQ304	45	0
**SCM3**	**WPM**			**0.1**	**NJQ305**	**45**	**1**
SCM4	WPM		0.1		NJQ306	45	0
SCM5	DKW			0.1	NJQ307	45	0
**SCM6**	**DKW**			**0.2**	**NJQ308**	**45**	**1**
SCM7	DKW			0.4	NJQ309	45	0
SCM8	DKW		0.1		NJQ310	45	0
SCM9	DKW		0.2		NJQ311	45	0
SCM10	DKW		0.4		NJQ312	45	0
**SCM11**	**DKW**	**0.1**		**0.4**	**NJQ313**	**45**	**1**

MS, Murashige and Skoog basal medium; WPM, woody plant basal medium; DKW, Driver and Kuniyuki walnut basal medium; 6-BA, 6-Benzylaminopurine; IBA, Indole-3-butytric acid; NAA, 1-Naphthylacetic acid.
The SCMs were added with 30g/L sucrose and gelled with 3 g/L gelrite, pH 5.8-6.0.

Supplementary Table 4. Callus induction frequency for NJQ305, NJQ308, and NJQ313 stem segments.

***C. bungei* half-sib families**	**Stem segment callusing medium**	**Frequency (%)**	**Significance***	**Embryogenic callusing medium**	**Frequency (%)**	**Significance***
NJQ305	SCM3	97.22±2.55	a	SCM3	39.89±3.19	a
NJQ308	SCM6	83.06±3.37	b	SCM6	29.51±1.90	b
NJQ313	SCM11	93.16±3.60	a	SCM11	36.99±2.38	a

The SCMs were added with 30g/L sucrose and gelled with 3 g/L gelrite, pH 5.8-6.0.
45 stem segments of NJQ305, NJQ308 and NJQ313 were were inoculated on SCM3, SCM6 and SCM11 respectively, repeated three times.
* Different lowercase letters (a and b) indicate the statistical differences between NJQ305, NJQ308, and NJQ313 ($P < 0.05$).

Supplementary Table 5. Composition of medium tested for differentiation and regeneration from embryogenic calli of *Catalpa bungei*.

Differentiating medium	Components					Frequency (%)
	Basic medium	6-BA (mg/L)	NAA (mg/L)	KT (mg/L)	ZT (mg/L)	
DM1	MS					0
DM2	MS	0.50		0.20		0
DM3	MS	1.00		0.20		0
DM4	MS		0.05	0.50	0.10	0
DM5	MS	0.60	0.15		0.10	0
DM6	MS	0.60	0.20		0.10	0
DM7	MS	0.60	0.40		0.10	0
DM8	MS	0.60	0.15		0.05	0
DM9	MS	0.60	0.15		0.15	0
DM10	MS	0.60	0.15		0.20	0
**DM11**	**DKW**	**0.60**	**0.15**		**0.20**	**100 ±0.00**

MS, Murashige and Skoog basal medium; DKW, Driver and Kuniyuki walnut basal medium; 6-BA, 6-Benzylaminopurine; NAA, 1-Naphthylacetic acid; KT, kinetin; ZT, zeatin.

The DMs were added with 30g/L sucrose and gelled with 3 g/L gelrite, pH 5.8-6.0.

30 embryogenic calli clumps were inoculated for each type of DMs, and repeated three times.

Supplementary Table 6. Composition of medium tested for shoot cutting rooting of *Catalpa bungei*.

Rooting medium	Components			Rooting frequency (%)	Significance*
	Basic medium	IBA(mg/L)	NAA(mg/L)		
RM1	1/2MS			0	f
RM2	1/2MS	0.01		78.89 ±5.09	b
RM3	1/2MS	0.05		83.33 ±5.77	a
RM4	1/2MS	0.10		86.67 ±6.67	a
RM5	1/2MS		0.01	41.11 ±6.94	e
RM6	1/2MS		0.05	55.56 ±3.85	d
RM7	1/2MS		0.10	75.56 ±6.94	c
RM8	1/2MS	0.10	0.10	87.66 ±3.75	a
**RM9**	**DKW**	**0.10**	**0.10**	**90.88 ±4.05**	**a**

1/2MS, half-strength MS; DKW, Driver and Kuniyuki walnut basal medium; IBA, Indole-3-butytric acid; NAA, 1-Naphthylacetic acid.
The RMs were added with 20g/L sucrose and gelled with 3 g/L gelrite, pH 5.8-6.0.
30 shoots were inoculated for each type of RMs, repeated three times.
* Different lowercase letters (a, b, c, d, e and f) indicate the statistical differences between RMs ($P < 0.05$).

Supplementary Table 7. Effect of *Agrobacterium* concentration.

Effect factors				Transformation frequency (%)	Significance*
***Agrobacterium* strain**	**OD_{600} value of *Agrobacterium* resuspesions**	**Infection duration**	**Co-cultivation time**		
EHA105	**0.2**	20 min	48 h	27.53±5.19	d
EHA105	**0.4**	20 min	48 h	80.25±5.35	a
EHA105	**0.5**	20 min	48 h	92.03±2.06	b
EHA105	**0.6**	20 min	48 h	97.65±2.04	a
EHA105	**0.8**	20 min	48 h	37.13±4.04	c
EHA105	**1.0**	20 min	48 h	17.93±3.58	e

DM11 was the best-selected medium for shoot regeneration, which is listed in Supplementary Table 5.

30 transformed embryogenic callus clumps were inoculated on DM11 containing 150mg/L kanamycin for each experiment, and repeated three times.

* Different lowercase letters (a, b, c, d, and e) indicate the statistical differences between different bacterial concentrations ($P < 0.05$).

Supplementary Table 8. Effect of co-cultivation time.

Effect factors				Transformation frequency (%)	Significance*
***Agrobacterium* strain**	**OD_{600} value of *Agrobacterium* resuspesions**	**Infection duration**	**Co-cultivation time**		
EHA105	0.6	20 min	**24 h**	91.86±1.95	a
EHA105	0.6	20 min	**48 h**	95.52±1.89	a
EHA105	0.6	20 min	**72 h**	57.65±2.72	b

DM11 was the best-selected medium for shoot regeneration, which is listed in Supplementary Table S5.
30 transformed embryogenic callus clumps were inoculated on DM11 containing 150mg/L kanamycin for each experiment, and repeated three times.
* Different lowercase letters (a, and b) indicate the statistical differences between different co-cultivation times ($P < 0.05$).

Supplementary Table 9. Effect of *Agrobacterium* strains and infection duration.

Effect factors				Transformation frequency (%)	Significance*
***Agrobacterium* strain**	**OD_{600} value of *Agrobacterium* resuspesions**	**Infection duration**	**Co-cultivation time**		
**EHA105**	0.6	**10 min**	48 h	70.54±6.47	c
**EHA105**	0.6	**20 min**	48 h	95.39±1.79	a
**EHA105**	0.6	**30 min**	48 h	77.16±4.10	bc
**GV3101**	0.6	**10 min**	48 h	27.78±5.09	d
**GV3101**	0.6	**20 min**	48 h	71.60±5.66	c
**GV3101**	0.6	**30 min**	48 h	83.80±3.33	b

DM11 was the best-selected medium for shoot regeneration, which is listed in Supplementary Table 5.
30 transformed embryogenic calli clumps were inoculated on DM11 containing 150mg/L kanamycin for each experiment, and repeated three times.
* Different lowercase letters (a, b, c, and d) indicate the statistical differences between different combinations of *Agrobacterium* strain and infection duration ($P < 0.05$).

Supplementary Table 10. Transformation efficiency of *Catalpa bungei*.

Replicates	No. of detected plants	No. of GUS positive plants	Positive frequency (%)	Average positive frequency (%)
1	103	95	92.23	
2	35	34	97.14	
3	26	22	84.62	92.31 ±4.92
4	25	24	96.00	
5	95	87	91.58	

Supplementary Table 11. The summary of sequence data from next generation sequencing.

**Samples**	**Clean reads**	**Clean bases (Gb)**	**Depth (×)**	**GC (%)**	**Q20 (%)**	**Q30 (%)**
WT	151,906,680	22,684,883,644	29.72	36.27	97.58	92.39
#2	149,360,140	22,325,631,422	29.24	39.29	97.69	92.84
#3	130,147,192	19,453,884,878	25.48	40.51	97.39	91.83
#7	160,671,490	24,004,434,282	31.44	36.47	97.85	93.20

Supplementary Table 12. The junction reads obtained by next generation sequencing in the genome of transgenic and wild-type *C.bungei*.

Plant	Position (s) in the reference genome	gDNA flanking the insert	Inserted T-DNA: position (s) in T-DNA vector	Estimated insert size (bp)
WT	--	--	--	--
#2	Group 4: 25,512,704	Left side	LB: 1,339-1,395	At least 1,395
#3	Group 1: 2,451,212<br>Group 6: 25,511,359	Right side	RB-CaMV poly (A) signal: 4875-5049	At least 175
#3	Group 0: 1,663,775	Left side	LB-Lac operator (1,204-1,220)+ Lac promoter (1,228-1,258)	At least 1,258
#7	Group 3: 21,586,673	Left side	LB- Lac promoter (1,228-1,258) + CAP binding site (1,273-1,294)	At least 1,294
#7	Group 5: 32,706,411	Left side	LB: 1,354-1,395	At least 1,395

-- means that no junction read was detected in the wild-type genome.
