# Supplementary material for: Efficient Transformation of Catalpa bungei Shows Crystal Genes Conferring Resistance to the Shoot Borer Omphisa plagialis
Source: Front Plant Sci. 2021 Dec 24;12:777411. doi: 10.3389/fpls.2021.777411 (PMC8739885; doi:10.3389/fpls.2021.777411)
Supplement: Supplementary file 2 [file Data_Sheet_2.pdf]

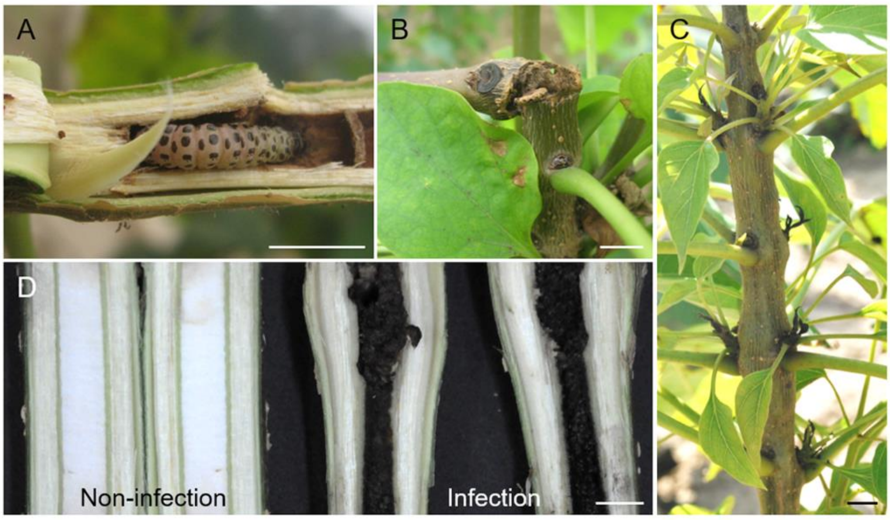

Supplementary Figure 1. Damage of *Catalpa bungei* by *Omphisa plagialis.*
(A) The larvae of *O. plagialis* bore into the pith of *C.bungei* shoots and damage them. The damaged shoots are easy to be withered and wind broken (B), and the damaged sites would form galls, and more side-branches (C). (D) The longitudinal sections of uninvaded and invaded shoot stems. Bars = 1 cm (A–D).

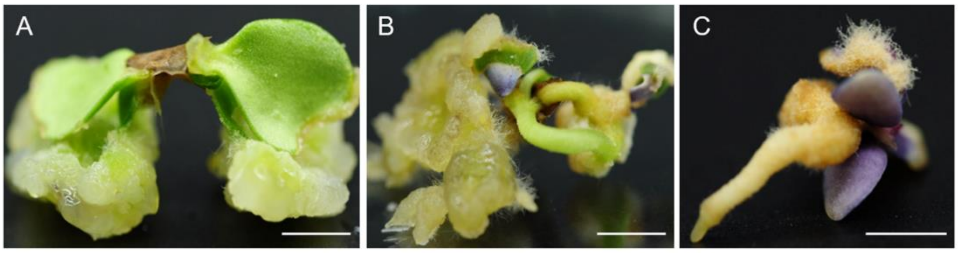

Supplementary Figure 2. High sucrose concentration was unfavorable for primary callus induction in *Catalpa bungei*.
(A) 30 g/L sucrose; (B) 60 g/L sucrose; (C) 90 g/L sucrose. Bars = 1 cm (A–C).

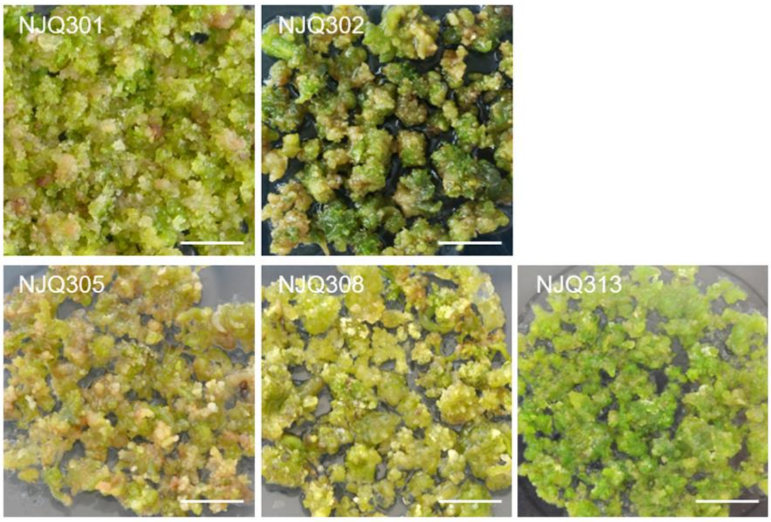

Supplementary Figure 3. Phenotypes of five types of embryogenic calli. Bars = 1 cm.

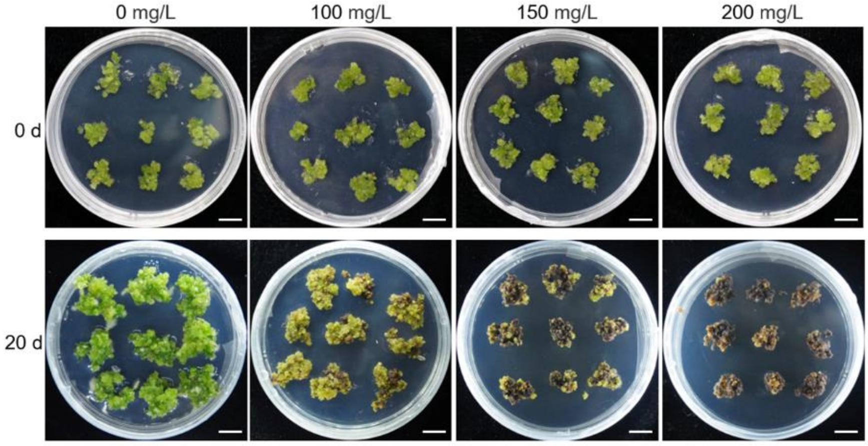

Supplementary Figure 4. Optimal concentration of kanamycin for selecting potential transgenic embryogenic calli.

The embryogenic calli turned brown after culturing for 20 d in DM11 with different concentration of kanamycin. Bars = 1 cm.

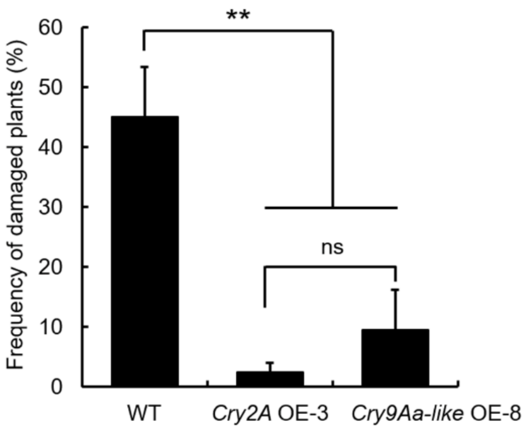

Supplementary Figure 5. Statistics on the frequency of damaged plants by *Omphisa plagialis* of wild-type and transgenic lines in the field.

Results are presented as means and standard errors from 60 wild-type (WT), 38 *Cry2A* OE-3, and 56 *Cry9Aa-like* OE-8 transgenic plants, ** $p < 0.01$, ns indicates no significant difference (Student's *t*-test).

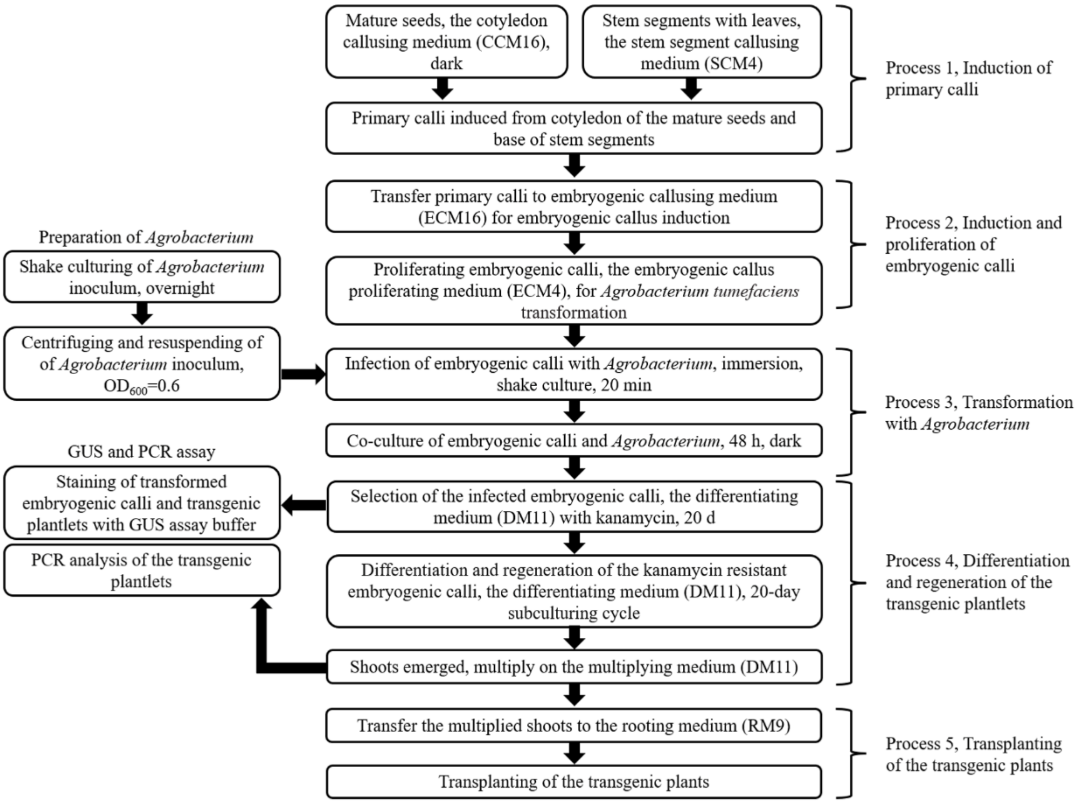

Supplementary Figure 6. Schematic diagram for *Agrobacterium*-mediated transformation of *Catalpa bungei.*
